# Could Artificial Intelligence guide surgeons’ hands?

**DOI:** 10.1590/0100-6991e-20233696EDIT01-en

**Published:** 2023-11-23

**Authors:** Jennifer A. Eckhoff, Ozanan Meireles

**Affiliations:** 1 - Harvard Medical School, Surgical Artificial Intelligence and Innovation Laboratory, Massachusetts General Hospital - Boston - MA - Estados Unidos

## EDITORIAL

In the rapidly evolving landscape of medicine and, in particular, surgery, the integration of Artificial Intelligence (AI) into clinical practice is no longer a futuristic fantasy but an unfolding reality. The potential of AI to revolutionize surgery lies not just in its capacity to process large amounts of data from various sources in record time, but in its ability to augment humans’ perception of the operative field and enhance surgical decision-making. The question, “Could AI guide surgeons’ hands?” is not merely rhetorical but a profound inquiry into the future of surgical care. While promises of enhanced procedural efficiency, intraoperative risk mitigation and navigation, and automation of repetitive tasks are highlighted in nearly every scientific paper focused on AI in surgery, key obstacles need to be overcome before this exciting new tool can be embraced in the day-to-day life of the operating room^1^. This editorial explores these obstacles while emphasizing the critical role of interdisciplinary discourse fostered by surgical societies. Additionally, we invite the Brazilian surgical community to shape the future of surgical AI by participating in an international, collaborative project, the Critical View of Safety (CVS) Challenge, endorsed by the Society of American Gastrointestinal Surgeons (SAGES).

Surgery has witnessed a paradigm shift with the advent of AI and machine learning. After the introduction of antiseptics, general anesthesia, and laparoscopy, AI is frequently referred to as the next surgical revolution. Novel technologies, predominantly based on surgical data science, including augmented reality, simulation, robotics, and computer vision-based analysis of operative video and imaging data, promise to enhance surgical precision, improve patient outcomes, and even potentially mitigate human error^2^. But the path to integrating these technologies into surgery is not without challenges. Foremost among these is the need for large quantities of high-quality data, curated in accordance with privacy regulations and labeled with respect to clinically relevant target features. In the realm of AI, this data is used to train algorithms to detect and predict such relevant features, and later be able to detect or predict the presence in unlabeled data (supervised machine learning). The development of robust, diverse, and generalizable AI algorithms, capable of providing consistent and reliable predictions regardless of intraoperative variety, requires data reflective of the diversity present in the real-world population^3^ ^,^ ^4^.

Most currently published work in the field of surgical AI, centered around the spatial analysis of the operative field, including instruments and anatomy, or temporal interpretation of surgical workflow, phases, steps, actions, and events^6^, is limited to locally acquired and curated datasets. The generalizability of the presented AI models, characterized by the ability to function similarly in other datasets from the same source procedure, is questionable. This means algorithms developed on a single dataset of one procedure from a single institution may not yield the same performance, measured by commonly established metrics such as accuracy and precision, in a dataset of the same procedure from another institution. To achieve this, the underlying training data has to mitigate the variations among these data sources, which can only be achieved through the composition of collective, global datasets. Surgical data challenges may present a solution in the quest for such comprehensive datasets^7^ ^,^ ^8^.

A sterling example of such an endeavor is the SAGES CVS Challenge. This initiative addresses an internationally established surgical safety measure in laparoscopic cholecystectomy - one of the most commonly performed, highly standardized procedures worldwide, that has become the benchmark in surgical AI. The proper achievement of the critical view of safety is a key step in preventing bile duct injury, therefore presents a clinically meaningful target. Beyond that, the CVS consists of three visually distinct features or criteria, that are a realistic target for computer vision-based classification. The SAGES CVS challenge has assembled a global dataset of approximately 1000 videos of the procedure from over 50 countries across the world. This data spans diverse patient groups, surgical approaches and techniques, surgeons’ expertise and style, technical and optical characteristics, creating an unprecedented resource for training AI models.

By crowdsourcing data acquisition and consecutive annotation, data challenges create an ecosystem of innovation, fostering the development of AI tools that are more accurate, reliable, and generalizable. Within this ecosystem, it is essential to address ethical considerations of inclusivity, fairness, equity, and bias at different stages of AI development and simultaneously comply with internationally variable privacy regulations. The SAGES data donation portal, developed by Surgical Safe Technologies, allows for easy use, worldwide access, and secure deidentification, while providing the opportunity to provide select, demographic metadata. The resulting dataset’s global nature ensures that the AI models trained on it are not narrowly focused on a specific demographic or set of clinical practices. This diversity is crucial for developing AI tools that can guide surgeons’ hands. Thus far, the Brazilian surgical community ranks second in the global data donation effort, behind the United States and just ahead of Australia and Canada, and data collection is ongoing. Yet, the journey of AI to clinical deployment is intricate. It involves not just the assembly of large, diverse datasets and subsequent development of algorithms. The meticulous annotation of these datasets, rooted in protocols and frameworks based on expert consensus, is paramount for the clinical relevance and applicability of the resulting AI. Despite the increasing availability of less supervised technology, the training and, more importantly, in the high-stakes context of surgery, the rigorous validation depends on high-quality labels to ensure they meet the subsequent AI meets the highest standards of accuracy and reliability.

The necessary infrastructure, as embraced by the SAGES CVS Challenge, entails a clinical expert consensus-based annotation protocol and structured annotator training. In its current state, annotator training, still welcoming annotators from across the world, targets surgical residents and fellows and provides a concise overview of the annotation prerequisites for high-quality machine learning development. The training entails a concise onboarding call with an experienced annotator, followed by a proficiency-based progression annotation of select data. The annotators’ performance is rated through interrater agreement with a predefined ground truth. This step-up approach ensures consistency and robustness in annotation9. The deployment of AI in the operating room must be based on sound, standardized surgical practices and governed by principles that prioritize patient safety to provide a collective surgical expertise to individual surgeons. While AI can guide and assist, the final decision-making authority must always reside with the human surgeon, who brings irreplaceable experience to the operating table. Therefore, the integration of AI into surgery also necessitates a shift in surgical training. Surgeons need to be equipped not only with technical skills but also with the knowledge to effectively interpret and utilize AI-generated insights. This includes understanding the capabilities and limitations of AI tools, as well as maintaining critical skills in situations where AI support may be limited or unavailable.

Beyond an infrastructure for collective, global dataset composition and annotation, the SAGES CVS Challenge provides a platform for interdisciplinary collaboration, fostering awareness of the requirements of surgical AI among surgeons as well as computer scientists. The road to fully integrating AI into surgical practice is laden with challenges but also brimming with opportunities, which can only be embraced through this form of interdisciplinary discourse, bringing together surgeons, data scientists, ethicists, and patients to shape the future of AI in surgery^10^.

The goal is to create AI tools that surgeons can trust as reliable assistants in the operating room. The envisioned role of AI in surgery is not as a replacement for human surgeons but as a collaborative partner. AI can provide real-time analytics, risk assessments, and decision support, enhancing the surgeon’s.

## Disclaimer

For more information on the SAGES CVS Challenge and how to become involved please visit www.cvschallenge.org or contact jeckhoff@mgh.harvard.edu.
